# Locked out during COVID-19 lockdown—an online survey of relatives of people with psychotic and bipolar disorders in Norway

**DOI:** 10.1186/s12889-022-12625-y

**Published:** 2022-02-13

**Authors:** Sofie R. Aminoff, Erlend Mork, Elizabeth Ann Barrett, Carmen Simonsen, Wenche ten Velden Hegelstad, Trine Vik Lagerberg, Ingrid Melle, Kristin Lie Romm

**Affiliations:** 1 Early Intervention in Psychosis Advisory Unit for Southeast Norway, Division of Mental Health and Addiction, Oslo University Hospital, Oslo, Norway; 2 Norwegian Centre for Mental Disorders Research (NORMENT), Institute of Clinical Medicine, University of Oslo, Oslo, Norway; 3 TIPS Centre for Clinical Research in Psychosis, Stavanger University Hospital, Stavanger, Norway; 4 Institute of Social Studies Faculty of Social Sciences, University of Stavanger, Stavanger, Norway; 5 Norwegian Centre for Mental Disorders Research (NORMENT), Division of Mental Health and Addiction, Oslo University Hospital, Oslo, Norway

**Keywords:** COVID-19, Pandemic, Bipolar disorders, Psychotic disorders, Relatives, Contingency plans

## Abstract

**Background:**

Many relatives of people with psychotic and bipolar disorders experience a high caregiver burden normally. During the first COVID-19 lockdown, mental health services partly shut down in many countries. The impact on relatives is unknown.

**Aims:**

Explore how relatives of people with psychotic and bipolar disorders experienced changes in treatment and service availability for their family member during the first COVID-19 pandemic lockdown in the spring of 2020, and to what extent they perceived information and support to be satisfactory. To help guide future contingency plans, we were also interested in what relatives would prioritize in the event of a future crisis.

**Study setting:**

We distributed an anonymous Norwegian online survey inviting relatives of individuals with psychotic and bipolar disorders. We distributed the survey using social media, through snowball sampling, collecting both quantitative and qualitative data. The survey was available between May and June 2020. We used systematic text condensation to analyse qualitative data.

**Results:**

Two hundred and seventy-nine respondents replied, mostly mothers and partners. A majority experienced a reduction in health care for their family member. Most respondents did not receive any support during the lockdown. However, most found the information they received from the mental health services regarding their family members’ treatment as sufficient. The qualitative data analysis revealed that relatives experienced three major challenges: reductions in treatment for the family member; reduced organised daily activity for the family member; and an increased caretaker load. In the case of a future lockdown, they would prefer increased access to care compared with a normal situation; increased support for relatives; and enhanced information.

**Conclusions:**

Mental health services in Norway did not manage to meet the needs of patients with severe mental illness and their relatives during the first COVID-19 lockdown. To be better prepared, Norwegian mental health services should consider prioritising infrastructure to ensure access to care and support for both patients and relatives. Digital tools and telephone calls are generally well accepted as substitutes for face-to-face contact.

**Supplementary Information:**

The online version contains supplementary material available at 10.1186/s12889-022-12625-y.

## Background

During normal circumstances, the relatives of people with psychotic and bipolar disorders tend to carry a high burden of care [1–3]. In the beginning of the COVID-19 pandemic, there was significant uncertainty about the modes of virus transmission, and it was difficult for policy makers to determine how strict the precautions should be. Many European countries responded by closing the parts of mental health services that was not considered necessary for preventing serious acute exacerbation of illness or death; these precautions assumed that social distancing was the most effective means for controlling the spread of the virus [4].

Early in the COVID-19 pandemic there were reports of deteriorating mental health in the general population of several European countries [5–7]. In Norway, the general population did not appear to have an increase in diagnosed psychiatric disorders per se during the first 6 months of the pandemic [8]. However, unpublished observations from a Norwegian self-report survey distributed widely to reach people with psychotic and bipolar disorders soon after the first lockdown confirmed that there were subgroups among these patients that have become markedly worse during the lockdown (Barrett EA, Simonsen C, Aminoff SR, ten Velden Hegelstad W, Lagerberg TV, Melle I, Mork E, Romm KL: The COVID-19 Pandemic Impact on Wellbeing and Mental Health Difficulties in People with Psychotic and Bipolar Disorders, under review).

A few studies have explored the actual use of mental health services during lockdown worldwide, but paradoxically, despite potential deteriorating mental health and reduced mental health service capacity, the pressure on emergency care in China and England was reduced [9, 10]. Pignon et al. [11] found a 54% reduction in psychiatric emergency consultations during the first period of lockdown in France. One possible explanation for this discrepancy may be a higher threshold to contact mental health services and that the responsibility shifted from the health care system to the relatives. Over the past few decades, the importance of collaboration between mental health services and relatives has been emphasised [12–14]. There are clear positive outcomes from involving families, for example, in the treatment of first episode psychosis [15] and bipolar disorder [12, 16, 17]. However, the degree to which relatives are involved varies widely across countries and services, ranging from no involvement at all, through sporadic psychoeducational sessions to psychoeducational family work conducted in single or multifamily groups over several months or years [18]. However, implementing more systematic family work has been challenging because of geographical barriers and limited time and access to trained family therapists [1].

Except for one study exploring the priorities and needs of family members to people experiencing psychosis within the context of one acute inpatient unit in UK during the COVID-19 pandemic [19], there are no studies that have systematically investigated relatives’ experiences with access to mental health services in general during the COVID-19 pandemic in any country. Furthermore, we know little about the specific needs of relatives’ to persons with severe mental illnesses during a crisis [20].

In summary, the aims of the current study were to investigate to what extent the relatives of people with psychotic and bipolar disorders experienced changes in treatment and service availability for their family member during the first COVID-19 pandemic lockdown in the spring of 2020 and to what extent they perceived information and support to be satisfactory. In addition, to inform future contingency plans from the relatives’ perspective, we wanted to better understand what relatives deemed important for mental health services to prioritise during a crisis such as a lockdown.

### Governmental restrictions during the survey period

The Norwegian government declared the first COVID-19 pandemic lockdown on March 12, 2020, going to April 6, 2020. Restrictions were then gradually lifted over the next months. During the first lockdown, all schools and kindergartens were closed, and all employees able to work from home were ordered to do so. Most mental health institutions closed all outpatient service provision, except those considered necessary to avoid serious exacerbation of mental illness or life-threatening behaviour. In general, group therapies closed, and there were strict limitations in accessing the physical facilities. Private psychologists/psychiatrists’ offices closed, and GPs warned people from turning up at their office unless strictly necessary. There were, however, local differences in the COVID-19 regulations and local differences regarding the availability of digital platforms that could substitute for the lack of physical appointments.

## Methods

### Study design and ethics

We developed a survey with both fixed and open response alternatives directed towards relatives of people with psychotic and/or bipolar disorders. The relatives self-reported their relationship to either a person with psychotic- or bipolar disorder, or both. The study was an anonymous online survey in Norwegian consisting of 48 questions, of which 16 were relevant to the aim of the present article. It was underlined that the period of interest was from the beginning of the lockdown, i.e., March 12 2020, when the Norwegian society locked down until the day the individual answered the survey in May–June 2020.

### The survey is available in Additional file 1.

We distributed the survey using social media, such as Facebook and Instagram. We also used the information channels of the Norwegian Bipolar Association, Mental Health Norway and Mental Health Carers Norway. Finally, we contacted psychoeducational family networks in Norway and asked them to distribute the survey to their members. This can be described as snowball sampling, a type of convenience sampling to reach out widely to recruit people who are difficult to identify and reach.

Norway has a population of 5,385,000 inhabitants and consists of mixed urban and rural areas. We disclosed and discussed the survey with the regional ethics committee, REC south-east. The committee did not regard the project as medical or health professional research as understood by the law, so the project fell outside the provisions of the Health Research Act. Oslo University Hospital’s data protection officer approved the project.

### Procedure

People that defined themselves as relatives of people with psychotic and/or bipolar disorders were invited to answer the online survey during May to June 2020. Two respondents were excluded because they did not report being a relative of someone with either bipolar or psychotic disorder. Two hundred and seventy-nine respondents reported that they were related to a person with a) a bipolar disorder, b) a psychotic disorder or c) both, which is understood as either a schizoaffective or psychotic bipolar disorder. We use the term psychotic bipolar disorder to describe this last group. Throughout the text, the respondents are called relatives, but the group may also contain other carers and friends. We use the term ‘family member’ to refer to the person with the relevant mental disorder.

### Analysis

The questions with fixed response alternatives were handled as quantitative data in the analyses, and the open-ended questions were treated as qualitative data. We analysed the quantitative and qualitative data separately to allow for different perspectives to emerge.

For quantitative data, in the text, we report results in the percentage of respondents (n) who answered affirmative to some extent (ranging from ‘some’ to ‘much’ to ‘very much’) for a specific question. In the tables, we have combined those who reported ‘much’ or ‘very much’ compared to those who did not. The group differences in the quantitative data were explored using chi square tests. We used IBM SPSS Statistics, version 27 for quantitative data analysis.

The qualitative data were analysed according to the principles of systematic text condensation [21]. The analysis was conducted in four steps by three researchers (EM, KLR and SRA). (1) EM, KLR and SRA read all the answers to the open-ended questions to achieve an overall impression and look for preliminary themes related to our research questions. (2) We broke the text into manageable meaning units and organised related meaning units into code groups. (3) We condensed the meaning under each code group. (4) Finally, we developed an analytic text—or a synthesis—about each category relevant for the study and collected citations that matched the essence of the categories. EM, KLR and SRA reviewed the transcripts three or more times to ensure that the data were accurately represented and interpreted. We used NVivo (version 10; QSR International LLC) for steps 2 and 3 of the qualitative data analyses.

### Sample

There was *n* = 87 (31%) relatives to a family member with a psychotic disorder, *n* = 135 (48%) relatives to a family member with bipolar disorder and *n* = 57 (21%) relatives to a family member with psychotic bipolar disorder (*N* = 279). The most common respondent was a mother (43%, *n* = 121), followed by a partner (23%, *n* = 65), a sibling (12%, *n* = 34), a father (2%, *n* = 2) and other relationships (18%, *n* = 50). Twenty-nine percent (*n* = 80) had received some type of support for relatives before the pandemic, such as psychoeducational single- or multifamily groups for psychotic or bipolar disorder or other forms of psychoeducational sessions. In addition, 10% (*n* = 26) were relatives to people who started their first treatment within the last year, 14% (*n* = 38) had started treatment 1–2 years ago, and the majority—77% (*n* = 215)—had started treatment more than 3 years ago.

## Results

In the result section we present results in two parts; quantitative and qualitative findings from ‘Relatives experiences form the first COVID-19 lockdown in Norway’ and ‘Lessons to learn to future crises’ respectively.

### Part one: Relatives’ experiences from the first COVID-19 lockdown in Norway

Here, we are presenting quantitative data first, before we present the qualitative findings.

### Quantitative data; Relatives’ experiences from the first COVID-19 lockdown in Norway

Eighty percent (*n* = 224) of the respondents had family members in treatment at the time of the lockdown. Out of these, 81% (*n* = 181) reported some degree of reduction (some, much or very much) in health care provision for their family member. Regarding community health care, 67% (*n* = 187) of the family members had received support *before* the lockdown. Out of these, 78% (*n* = 146) experienced reduced access to support during the lockdown. Out of the respondents (*n* = 221) who answered questions about the information received from health care providers about treatment in the lockdown period, 58% (*n* = 128) reported that the information had been satisfactory to some extent. As outlined in Table 1, there were no significant differences between diagnostic groups in either treatment reduction, community health care support or the presence of satisfactory information.

**Table 1:**
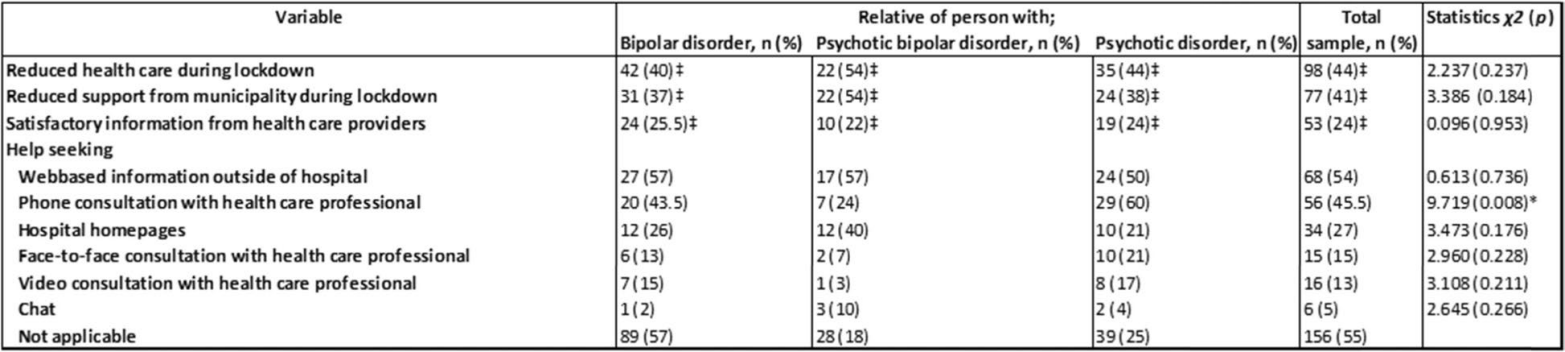
Relatives’ experience of health care provision and support (*N* = 279)

‡Percentage of those receiving treatment answering much/very much

*Significant results

Out of the 45% (*n* = 126) relatives who sought or received support, the most common form of support was visiting web pages outside of the health care system, (54%, *n* = 68). This was followed by phone consultations with health care professionals, (46%, *n* = 56), hospital web pages, (27%, *n* = 34), face-to-face consultations, (15%, *n* = 18), video consultations, (13%, *n* = 16) and internet chat options (4%, *n* = 6). Significantly fewer relatives of people with psychotic bipolar disorder had phone consultations with health care professionals compared with the other two groups. Otherwise, there were no significant differences between the groups (see Table 1).

### Qualitative data: Relatives’ experiences from the first COVID-19 lockdown in Norway

We extracted two main themes from the analysis of open-ended questions in which the relatives were asked to elaborate on their experiences during the lockdown. The main themes were as follows: Worsening for relatives during COVID-19 pandemic (Four subthemes) and Helpful for relatives during COVID-19 pandemic (one subtheme) (For more details, see Table 2).

**Table 2:**
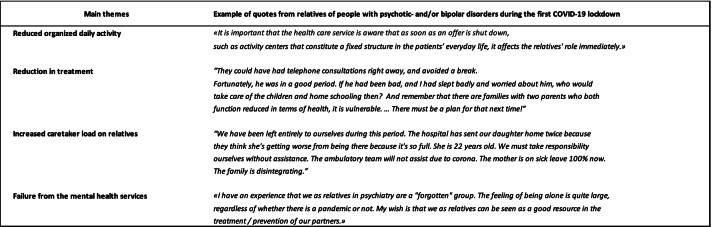
Relatives’ quotes about experiences during COVID-19 lockdown

### First main theme: Worsening for relatives during COVID-19 pandemic

Four subthemes emerged from the first main theme; reduction in treatment; reduction in organised daily activities; increased caretaker load; and little help from mental health service (see Table 2).

### Reduction in treatment

Reports reflected experiences of mental health services, jobs, schools, universities, social and employment services and other activities in the local community were reduced or completely shut down. Contact with public services, such as social and employment services was also more difficult.

Several relatives reported that it had been more difficult to receive inpatient treatment during the lockdown period. One described the following:

*He was refused hospital admission when he needed it at the beginning of the corona lockdown. He had to go 14 days in a state of severe mania that turned into psychosis, ending with the police and involuntary hospitalisation.*

The systematic text condensation is presented in syntheses in Table 3.

**Table 3:**
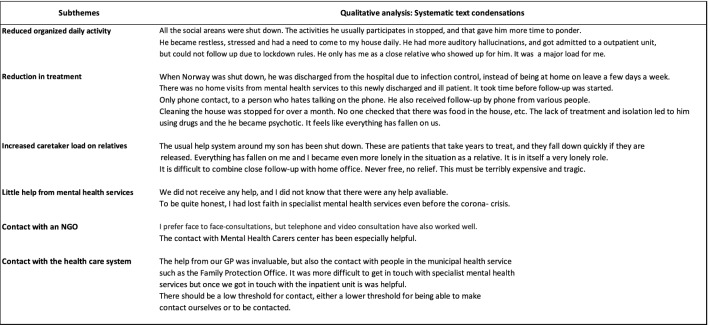
Syntheses from systematic text condensation

### Reduced organised daily activity

Several relatives noted both the lack of work and activity, with more time for rumination, as being a negative consequence of the lockdown for their family member. In addition, several described that a lack of daily routines had negative effects (See Table 3 for synthesis).

### Increased caretaker load

Relatives pointed out that in a demanding situation like a pandemic, there is a need to increase access to care for this patient group rather than the opposite. The relatives experienced that they constituted the only support system when everything else was locked down. They took on many tasks during the lockdown that would usually be carried out by mental health and community services, ranging from cleaning and grocery shopping to social support and treatment tasks. A mother describes herself as a *‘…mother, doctor, psychologist, infection control officer, friend and more’.* (See Table 3 for synthesis)*.*

### Little help from mental health services

There were reports from relatives who had not received any help, and/or they described a lack of confidence in the mental health services already before the COVID-19 crisis set in. One relative expressed this quite simply: *‘I have not been offered help or received information about available help’.* (See Table 3 for synthesis)*.*

### Second main theme: Helpful for relatives during COVID-19 pandemic

This main theme had two subthemes which was the importance of contact with a nongovernmental organisation (NGO), or the health care services.

### Contact with an NGO

Several relatives highlighted contacting nongovernmental organisations for relatives (e.g., *Mental Health Carers Norway*) as particularly helpful. (See Table 3 for synthesis).

### Contact with the health care system

Some mentioned support from professionals in community health services, such as GPs. From the relatives’ perspective, it appears to have been more difficult to get in touch with specialist mental health services than community services during the lockdown. (See Table 3 for synthesis).

### Part two: Lessons to learn for future crises

The second part of the results section deals with the respondent’s priorities for future crises, and may help guide future mental health contingency plans.

### Quantitative data: Lessons to learn for future crises

Most of the participants (*n* = 226; 81%) responded to the question about what types of communication platforms they thought the mental health services should prioritise for relatives in the event of a similar situation in the future. The most common response was to prioritise phone calls (50%), followed by face-to-face consultations (47%), video consultations (45%), chat (36%), web-based information (27%) and better homepages for hospitals (17%). There were no differences between the groups, save for the item of the use of chat rooms being significantly more valued by relatives to people with bipolar disorder alone compared with those with family members with psychotic symptoms (the other two groups), see Table 4.

**Table 4:**
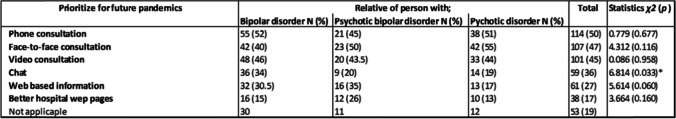
Relatives suggestions for future prioritisation during crises (*n* = 226)

*Significant results

### Qualitative data: Lessons to learn for future crises

When the relatives were asked what should be prioritised for future crises, such as a lockdown, the qualitative analysis revealed three subthemes: access to care, increased support and information to relatives (see Table 5).

**Table 5:**
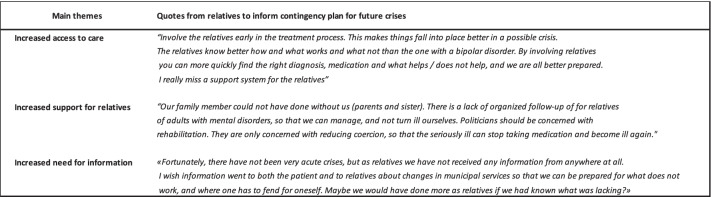
Quotes from relatives about the contingency plans for mental health services

### Access to care

The message from many of the relatives was that facilitating cooperation between the mental health services and relatives is particularly important in a crisis, such as the COVID-19 lockdown. Many commented that they had rarely—or never—experienced the health care service contacting them during this period, and they had been responsible for the initiation of all the contact themselves. When social services shut down, the relatives become even more involved.

"*Do not shut down. Continue or preferably increase the services to the patients and their relatives. This is especially important for those in an early stage of illness*".

### Increased support

Many relatives described a strong sense of standing alone. Some emphasised that the mental health services must be proactive and reach out to relatives. Several found it useful to be invited in during video consultations between the family members and their clinician. The relatives expressed that they would feel safe if they knew they could get in touch and get help when needed. The relatives were open to different types of communication, including telephone, video and face-to-face talks.

### Information to relatives

A common comment was lack of information about major changes in the treatment of the family member. Several described that they did not receive information about how the mental health service worked during the lockdown.

One relative noted, *‘I did not know that the outpatient clinic did not contact my family member anymore, and therefore it took a few weeks before I understood why he got worse’.*

## Discussion

The main finding from the current study is that the relatives reported reductions in mental health services, community services and daily activities for their family members during the first COVID-19 lockdown and that this had a direct impact on their role as caregivers. Furthermore, the relatives reported little support from the mental health services and little help in aiming to empower them during this crisis. This lack of attention and support was described as an exacerbation of a problem present long before the crisis set in, reflecting an experience of limited attention from the mental health services concerning the relatives’ needs.

The relatives stressed the importance of the mental health services’ ability to use the available means to provide the necessary care and support in an emergency such as the COVID-19 lockdown. This implies a readiness to increase access to services when this is appropriate and to avoid serious exacerbation of illness. Increased access to support for relatives is important because their caregiver role is likely affected by any situation that reduces the capacity of the mental health services.

Our findings indicate that the mental health services in Norway were not properly prepared for a crisis involving a sudden reduced capacity to take care of people with psychotic and bipolar disorders over time. Actions such as increased support for relatives could be added systematically to the contingency plans for different types of disasters.

A review of previous emergencies and disasters, found that a major part of those who take an active role in emergencies are volunteers who improvise and assist where they discover a need [22]. One could consider the help provided by relatives as an extension of this form of volunteering. Indeed, relatives are often prepared to step up during a crisis because of sheer necessity. There is major potential for relatives to feel disempowered when viewed as a resource for services. Many relatives wished to have more involvement, particularly in a crisis, but there is a challenge in accommodating diverse needs in complex services. Family involvement requires a balance on the part of service leaders to support availability while also maximising decision making for patients and their relatives [23]. However, relatives are seldom supported and included as part of a contingency plan. We suggest that future work with contingency plans considers involving user organisations—particularly relatives—to look for resources and how these resources can be used and supported by the health care system during a crisis. This is in line with Onwumere and co-authors suggestion to recognise relatives (or informal carers) as hidden key workers in need of emotional, social, practical and financial support, advocating programmes that help relatives improve their situation [24].

In addition to strengthening carer organisations, we suggest three considerations for involving the resources of relatives into contingency plans.

### First, it is important to establish a relationship with relatives as early in the treatment as possible

Even though under normal circumstances relatives provide important care, they normally experience limited involvement in the treatment of their family member from the mental health services [25]. The experiences of lacking information, knowledge and support expressed by the relatives in the current study underlines the importance for mental health services to establish a relationship with relatives during normal times and as soon as possible after the initiation of treatment. Previous research has shown that health professionals often assume that all exchanges of information include breaking the strict rules of confidentiality [26, 27]. Indeed, confidentiality is challenging, and a crisis will not allow health professionals to ignore these rules. However, providing relatives with the information, knowledge and tools necessary to cope with psychotic and bipolar disorders can be accomplished without breaking the law of confidentiality. This can include general knowledge about these disorders, guidelines for treatment and information about who relatives should turn to if problems exceed what they feel they are able to cope with.

### Second, relatives are open to a variety of approaches, such as phone and/or video consultations, as acceptable substitutes for face-to-face contact

There has been a boom in mental health services’ willingness and infrastructure to use video consultations and modern technologies for substituting real-life contact during the pandemic [28, 29]. However, it is important to bear in mind that a phone call can be of great value for relatives, as indicated by the current study’s findings. The present study also indicates that it is essential that a low threshold for telephone contact be signalled as a minimum for relatives of people who are receiving care at the time of crises, such as a pandemic lockdown. This may be important for the relatives of both outpatients and inpatients, especially those having a family member involuntarily admitted to the hospital [30]. Another qualitative study interviewing carers of people admitted to inpatient care in one hospital due to psychosis during the COVID-10 pandemic also found that carers felt excluded from care. The study suggests that family members appreciate having good working relationships with inpatient staff, being informed about ongoing care and being able to keep in contact with their loved one even by remote means [19].

### Third, many relatives are caring for persons that are in and out of care

Because some patients have longer periods without will or capacity to receive care, their families are especially vulnerable during a crisis, such as the lockdown. For these relatives, contact with the mental health services will often be fragmented and even more difficult to navigate because of a lack of continuity. In these cases, the relatives will often be the only persons who can intervene if their family member experiences severe symptom exacerbation. These relatives should have easy access to qualified support, which should not be limited by their family members’ reluctance to receive care.

It is also worth drawing attention to the finding that a lower proportion of relatives of people with psychotic bipolar disorder had received any phone calls from mental health services during the pandemic compared with the other diagnostic groups. One possible explanation is that, in Norway, people with bipolar disorder with a propensity for psychotic symptoms are not admitted to outpatient units as often as patients with primary psychotic disorders but are rather treated by their GPs in between pronounced mood episodes. Consequently, exacerbations during a pandemic lockdown may result in more of their relatives having no link to specialised mental health care providers.

To support people with psychotic and bipolar disorders and their relatives during a crisis, it is vital to ensure the channels for communication between the affected person and their relatives and the mental health care providers remain open. Technology can offer such platforms; digital tools, telephone and chats may all be viable channels; the most important message is to ensure easy access to support and treatment. This shift toward telemedicine and technology use for caregiver integration may persist even after the pandemic [31]. Our study however suggests that relatives’ needs may vary according to the symptom profile of their family member, which is in line with other studies [32]. The current study reveals that a relative to a family member with bipolar disorder without psychotic symptoms may have different needs (for example may appreciate the possibility to chat with someone in the health care services), than relatives to someone with psychotic bipolar disorder who may have a greater need to talk to someone knowing how to handle psychotic symptoms.

### Strengths and weaknesses

Although we do not know whether our sample is representative for the entire Norwegian population of relatives of family members with psychotic and bipolar disorders, we have respondents from all health regions of Norway, and the distribution of respondents corresponds to a large extent with the population density in the health regions. We also have a relatively large percentage of respondents whose family members are not in treatment, and this group is usually not represented in studies of relatives. One limitation is that the survey was in Norwegian, hence excluding people who do not understand Norwegian. We did not ask about the age and gender of the respondents, which prevented us from investigating the influence of these characteristics. Because ‘mother’ was the most common response in the role category, it seems that our survey was biased regarding having more female respondents. This is in line with other online surveys [33]. Another limitation is that respondents self-reported their family members diagnosis, so we did not ascertain diagnosis systematically. Lastly, we did not determine the sample size before the study.

## Conclusion and clinical implications

In sum, many of the relatives of people with psychotic and bipolar disorders in Norway, experienced being left alone during the pandemic, with little support from mental health services. The relatives in the current study clearly expressed that they carried a large burden during the lockdown, which they believe could have been relieved with increased support and guidance via phone or other digital means.

Thus, future contingency plans should include measures of how to support relatives. For people not being in treatment, communication about access to adequate advice for relatives should be clearly presented on appropriate websites. Access to appropriate support should also be prioritised to the relatives of family members who are reluctant to receive care. It is time to step up and take care of the carers.

## Supplementary Information

**Additional file 1.** National COVID-19 relatives survey.

## Data Availability

Out of respect for the respondents, we do not wish to share our raw data publicly since it was not stated in the informed consent that raw data would be published. Some respondents shared sensitive stories. Therefore, the datasets used and/or analysed during the current study are available from the corresponding author on reasonable request.

## Abbreviations

COVID-19: Coronavirus disease of 2019
GP: General practitioner
NGO: Nongovernmental organisation
UK: United Kingdom
